# Impact of cetuximab plus cisplatin alone and cetuximab plus cisplatin and paclitaxel regimen on humanistic outcome in head and neck cancer

**DOI:** 10.1186/s43046-023-00160-9

**Published:** 2023-01-19

**Authors:** Avinash Khadela, Bhavin Vyas, Mustakim Mansuri, Dipen Sureja, Kunjan Bodiwala

**Affiliations:** 1 Department of Pharmacology, L. M. College of Pharmacy, Navrangpura, Ahmedabad, Gujarat 380009 India; 2 Department of Pharmacology, Maliba Pharmacy College, Uka Tarsadia University, Surat, Gujarat India; 3 Department of Pharmaceutical Chemistry, L. M. College of Pharmacy, Navrangpura, Ahmedabad, Gujarat 380009 India; 4 Department of Pharmaceutical Chemistry and Quality Assurance, L. M. College of Pharmacy, Navrangpura, Ahmedabad, Gujarat 380009 India

**Keywords:** Head and neck cancer, Quality-adjusted life-years, Cetuximab, Cisplatin, Paclitaxel, Recurrent or metastatic head and neck squamous cell carcinoma

## Abstract

**Background:**

The prevalence of head and neck cancer (HNC) is increasing rapidly, and the prognosis is poor in the advance stage. For the patient suffering from advance stage HNC, the improvement in quality of life and decrease mortality remain as the mainstay of treatment. The aim was to assess the change in quality-adjusted life-years (QALYs) in recurrent or metastatic HNC patients receiving cetuximab plus cisplatin and cetuximab plus cisplatin-paclitaxel.

**Methods:**

It was a single-centric prospective-observational study. Patients were divided into two cohorts based on the chemotherapy regimens they were prescribed. Patients in cohort 1 were prescribed with cetuximab and cisplatin and in cohort 2 were prescribed with cetuximab, cisplatin, and paclitaxel. The QALYs were the primary outcome of the study, and it was calculated using EQ-5D-5L instrument. Patients were followed until the completion of the therapy, i.e., six chemotherapy cycles. The statistical analysis was carried out using SPSS for descriptive and inferential analysis.

**Results:**

Amongst 175 patients screened, 100 patients were enrolled which further distributed in cohorts 1 and 2 equally. The mean QALYs were 0.016 and 0.017 at the time of diagnosis, i.e., before initiation of chemotherapy for patients in cohorts 1 and 2, respectively. At every chemotherapy cycle, the QALYs were calculated. After the completion of six chemotherapy cycles, the mean QALYs were 0.029 and 0.032 for patients in cohorts 1 and 2, respectively.

**Conclusion:**

The three-drug therapy consisting of cetuximab, cisplatin, and paclitaxel has shown significant improvement in patients’ QALYs compared to two-drug regimens of cetuximab and cisplatin. Thus, if the therapy consisted of three-drug regimen is used instead of two-drug regimen, it will have a positive impact on humanistic outcome in recurrent or metastatic HNC patients.

**Supplementary Information:**

The online version contains supplementary material available at 10.1186/s43046-023-00160-9.

## Background

The prevalence of head and neck cancer (HNC) is rapidly increasing, with more than 550,000 registered cases and more than 300,000 deaths annually [1]. Squamous cell head and neck carcinoma (HNSCC) is the most common type of HNC, which represents for 90% of HNC cases. Amongst various anatomical sites in the head and neck region, nasopharynx, oral cavity, larynx, and pyriform fossa are the common sites of HNC [2]. Patients diagnosed with advanced or recurrent (R/M) HNSCC have a poor prognosis, with a median survival duration of less than 1 year [3, 4]. The relapse rate is very high in HNSCC, and metastasis occurs in nearly 10% of cases. Thus, to monitor disease progression, enhance quality of life, and improve survival in patients suffering from HNSCC, complex chemotherapeutic regimens need to be prescribed.

Currently, advanced and recurrent HNSCC patients are being treated with anti-epidermal growth factor receptor (EGFR) cetuximab along with cisplatin or carboplatin and 5-flurouracil (5-FU) as a first-line treatment protocol as per EXTREME protocol [5–8]. This protocol has shown significant improvement in overall survival (OS) compared to previously used chemotherapy protocols without inclusion of cetuximab. Despite of having good efficacy, this protocol has shown deteriorating safety especially cardiac toxicities and other life-threatening adverse events [9–11]. This compromised safety could negatively affect the patient’s quality of life. As a result, alternative systemic chemotherapeutic protocol need to identify with better safety along with efficacy [11]. Numerous authors have reported that replacing 5-FU with taxanes could minimize the toxicities and improve patient’s quality of life [12, 13]. However, the impact of this protocol on patient’s quality of life using quality-adjusted life-years (QALYs) remains inexplicable.

Cetuximab is a monoclonal antibody that binds to the EGFR and inhibit the expression of tyrosine kinase [14]. Activation of tyrosine kinase is associated with progression of disease in patients suffering from HNSCC [15]. Thus, inclusion of cetuximab along with platinum and taxanes not only increases the efficacy but also increases the effectiveness of radiotherapy.

The QALYs are calculated to differentiate quantity along with quality of patients’ survival. The numerous methods are available to calculate QALYs, namely time trade-off, standard gamble, and visual analogue scale. However, EQ-5D-5L is the widely accepted tool provided by the EuroQoL group as it has been validated in numerous different patient population. It included major five dimensions which directly correlated with patients’ health-related quality of life such as mobility, ability to self-care, ability to undertake usual activities, pain and discomfort, and anxiety and depression [16]. Moreover, there are five levels which define the degree to which these five dimensions are altered or having an issue. Several authors have used QALYs to assess the impact of new treatment options in comparison with conventional therapy and its worthiness, safety, and efficacy in cancer patients and to assess and determine the patients’ preferences for health states [17–21]. Amongst numerous methods available for the calculation of QALYs, EQ-5D-5L is a widely used instrument all across the globe [16, 22]. The calculated QALYs can be correlated with health economics such as total healthcare cost and direct and indirect costs to justify the cost-effectiveness or superiority of regimens considering humanistic and economic outcomes.

This study was designed to illustrate the change in patient’s QALYs in recurrent or metastatic head and neck cancer patients receiving cetuximab plus cisplatin alone or cetuximab plus cisplatin and paclitaxel.

## Methods

### Study design

This was a prospective, single-centered observational study conducted for the period of 2 years.

### Ethical approval

The study was approved by the Institutional Ethics Committee of Maliba Pharmacy College, Bardoli (reference number MPC/HREC/01/2017–18; dated 21 December 2017).

### Setting and inclusion/exclusion criteria

The study was conducted at Bharat Cancer Hospital, Surat, India.

#### Inclusion criteria

The inclusion criteria were as follows: (1) patients having age between 18 and 80, (2) R/M HNSCC patients who were not previously treated, and (3) patients with an Eastern Cooperative Oncology Group (ECOG) performance status (PS) of 0 or 1.

#### Exclusion criteria

Patients receiving only radiation therapy, not willing to sign informed consent form, prescribed with other than these two regimens and pregnant women, were excluded from the study.

### Patient enrollment

Patients with confirmed diagnosis of HNSCC were recruited from a single oncology setup. Patients were recruited into two cohorts: cohort 1 and cohort 2. Patients recruited in cohort 1 were prescribed with cetuximab plus cisplatin regimen, and patients in cohort 2 were prescribed with cetuximab plus cisplatin plus paclitaxel.

### Measurement of humanistic outcome

The QALYs were calculated using EQ-5D-5L technique in both groups at certain pre-determined intervals. Patients were asked to fill the questionnaire consisted of five dimensions which determine overall quality of life, namely mobility, self-care, usual activities, pain/discomfort, and anxiety/depression. This questionnaire was filled by the patients before initiation of chemotherapy and then after receiving every chemotherapy cycle until the completion of 5 cycles. The QALYs are then calculated using utility index and years lived (time period between initiation of the chemotherapy until the completion of therapy). Adverse events were noted at every visit and analyzed as per the National Cancer Institute — Common Terminology Criteria for Adverse Events, version 5.0.

### Data analysis

The sample size of 90 patients, 45 in each cohort, is sufficient to detect a clinically significant difference of 0.5 amongst both the cohorts. Considering the dropout, 100 patients, 50 in each cohort were recruited. The eligible patients were randomized amongst the two study cohorts based on a computer-generated sequence using opaque envelop technique by independent drug dispenser. To conceal the randomization sequence, we used sequentially numbered, opaque sealed envelopes. Descriptive analysis and inferential analysis were carried out using SPSS (trial version).

## Results

Amongst 175 patients screened, a total of 100 patients complaining the inclusion and exclusion criteria were included in the study. The 75 patients who were excluded from the study were dropout or not willing to participate in the study. From these 100 patients, 50 patients were allocated to cohort 1 and 50 were to cohort 2. The patients’ baseline demographic and disease-related characteristics were mentioned in Table 1. The majority of patients’ age were ranging between 61 and 70 years and were male. The family history was found absent in 76% and 82% patients in cohorts 1 and 2, respectively. Hypertension and diabetes were common comorbidities, and oral cavity followed by oropharynx, hypopharynx, and nasopharynx was common anatomical site of R/M NSCC. Out of 100 patients, 31% patients had metastasis, and 21% had local or regional recurrence.

**Table 1:** Patient’s baseline demographic and disease-related characteristics

Parameter	Cohort 1 (*n* = 50) No. of pts. (%)	Cohort 2 (*n* = 50) No. of pts. (%)
Age (years)		
31–40	01 (02)	02 (04)
41–50	08 (16)	12 (24)
51–60	18 (36)	11 (22)
61–70	22 (44)	18 (36)
71–80	01 (02)	07 (14)
Gender		
Male	45 (90)	43 (86)
Female	05 (10)	07 (14)
Family history		
Present	12 (24)	09 (18)
Absent	38 (76)	41 (82)
Anatomical site of tumor		
Oral cavity	28 (56)	33 (66)
Oropharynx	08 (16)	07 (14)
Nasopharynx	05 (10)	03 (06)
Hypopharynx	07 (14)	04 (08)
Larynx	02 (04)	03 (06)
ECOG PS		
0	28 (56)	26 (52)
1	22 (44)	24 (48)
Site of recurrence		
Local recurrence	07 (14)	09 (18)
Locoregional recurrence	11 (22)	12 (24)
Metastasis	16 (32)	15 (30)
Metastasis and local or regional recurrence	12 (24)	09 (18)
Regional recurrence	04 (08)	05 (10)
Comorbidities		
Hypertension	16 (32)	17 (34)
Diabetes mellitus	14 (28)	12 (24)
Asthma	08 (16)	06 (12)
Chronic obstructive pulmonary disease	02 (04)	04 (08)
Gastric ulcer	09 (18)	11 (22)
No comorbidities	01 (02)	00 (00)

Eastern Cooperative Oncology Group (*ECOG*), performance status (*PS*)

In both cohorts, the QALY was estimated during each visit. The initial QALY before the commencement of chemotherapy in cohorts 1 and 2 was 0.016 and 0.017. After the completion of 5 cycles of chemotherapy, the QALY in cohorts 1 and 2 was 0.029 and 0.032. The detail QALY at each visit has been mentioned in Table 2.

**Table 2:** comparison of quality-adjusted life-years amongst cohort 1 and cohort 2 in recurrent or metastatic head and neck cancer patients

Group	Visit	Mean ± SEM (QALYs)
Cohort 1	1	0.016 ± 0.001
	2	0.014 ± 0.001
	3	0.019 ± 0.001*#
	4	0.026 ± 0.001*#$
	5	0.025 ± 0.001*#$
	6	0.029 ± 0.001*#$@!
Cohort 2	1	0.017 ± 0.001
	2	0.016 ± 0.001*
	3	0.021 ± 0.001*#
	4	0.027 ± 0.001*#$
	5	0.032 ± 0.001*#$@
	6	0.038 ± 0.001*#$@!

*P < 0.05, compared to V1 of respective group, #compared to V2 of respective group, $compared to V3 of respective group, @compared to V4 of respective group, !compared to V5 of respective group. Data presented as mean ± SEM. Data analyzed by two-way ANOVA followed by Tukey’s multiple comparison test (*CI* = 95%, *P* < 0.05)

The majority of patients complained of grade 2 neutropenia followed by fatigue, nausea, oral mucositis, anemia, etc. Cardiotoxicities and treatment-related death were not observed throughout the study. All the adverse drug reactions occurred in both cohorts were mentioned in Table 3.

**Table 3:** The safety profile of patients suffering from recurrent or metastatic head and neck cancer

Sr. no.	Adverse drug reaction	Grade of ADR as per CTCAE system	Cohort 1 Incidence proportion (%)	Cohort 2 Incidence proportion (%)
1	Neutropenia	Grade II	12	11
2	Oral mucositis	Grade I	6	4
3	Peripheral neuropathy	Grade II	4	2
4	Fatigue	Grade II	9	7
5	Nausea	Grade I	8	6
6	Vomiting	Grade II	4	3
7	Weight loss	Grade I	3	2
8	Anemia	Grade II	7	4
9	Skin rash	Grade I	4	4

*ADR* adverse drug reaction, *CTCAE* version 5 common terminology criteria for adverse events. Data presented as percentage. Data analyzed by Fisher’s exact test followed by Tukey’s multiple comparison test (*CI* = 95%, *P* < 0.05)

**Fig. 1:**
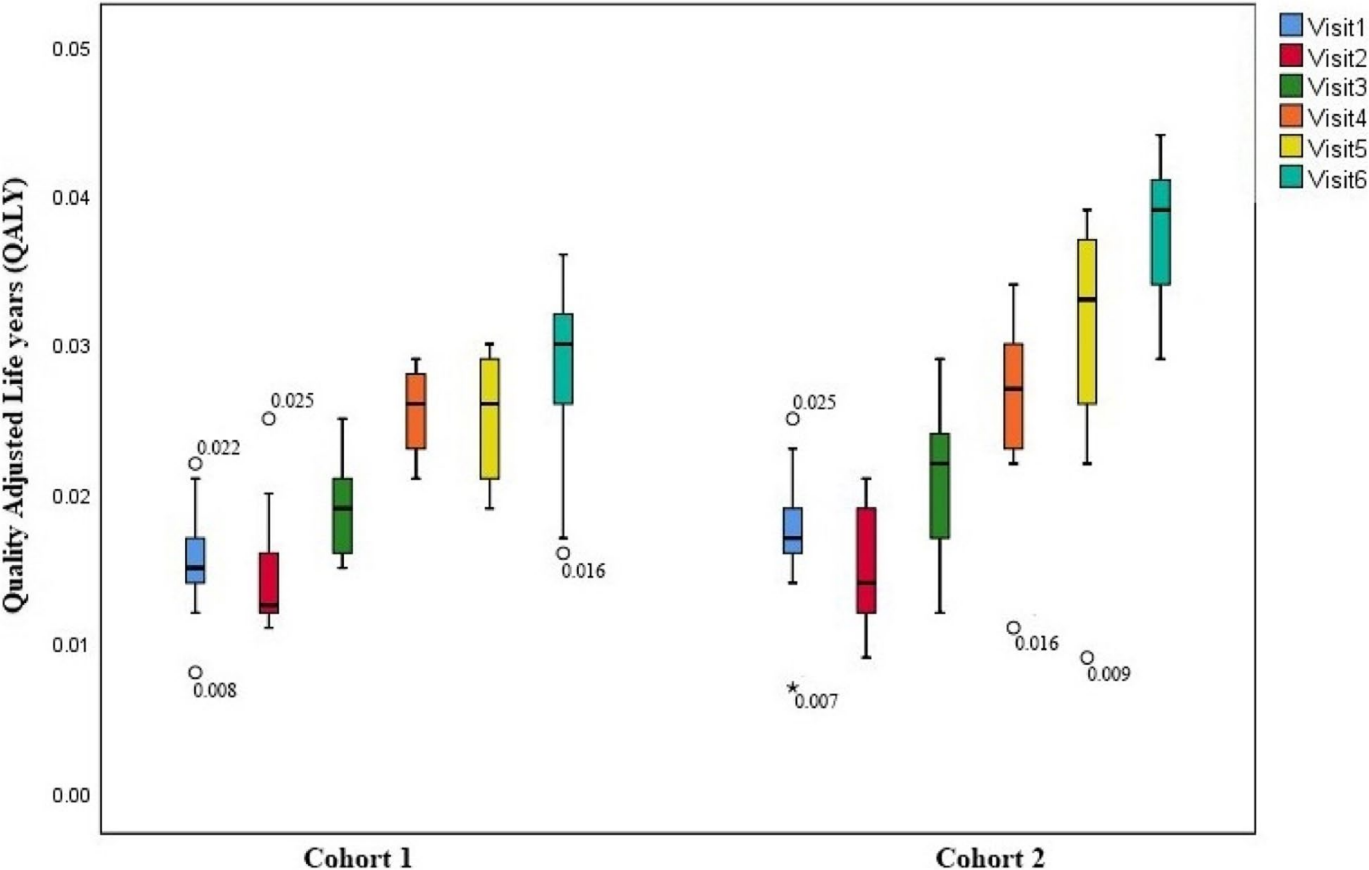
The trend of QALYs in patients suffering from recurrent and metastatic head and neck cancer in cohort 1 and cohort 2 at pre-determined intervals

The box-whisker plot shown in Fig. 1 reveals the skewness and distribution of QALYs data. Each box contains the median, lower, and upper quartile values, as well as the minimum and maximum range of QALYs. In cohort 1, the QALYs significantly changed from 0.016 to 0.029, whereas in cohort 2, it changed from 0.017 to 0.032. The cohort 2 has shown significantly better QALYs compared to cohort 1 at the end of therapy. The number highlighted in the box-whisker plot indicated legitimate outliers.

## Discussion

In this study, an attempt was made to compare and analyze the QALYs in patients suffering from R/M HNSCC receiving cetuximab and cisplatin alone and cetuximab, cisplatin, and paclitaxel combination. We found that patients who are prescribed with three-drug regimen compared to two-drug regimen have reported better quality of life. This may be due to higher efficacy and lesser toxicity of this three-drug regimen [23].

Majority of the patients in this study were having age between 60 and 70 years as the incidence of HNC increases with age [24]. Considering the social habits, namely smoking, chewing tobacco or any other form of tobacco consumption is responsible behind the higher prevalence of HNC in males [25]. In this study, we have reported 90% and 86% male patients in cohorts 1 and 2, respectively [26]. Family history is considered as insignificant factor in determining the risk factors of HNC. Similar findings were observed in our study with 76% and 82% cases in cohorts 1 and 2, respectively, were having no family history of HNC. The prevalence of oral cavity HNC is highest amongst other anatomical sites of HNC in our study which might be attributed to the social habits such as tobacco chewing or smoking [27]. We found that majority of patients were suffering from hypertension or diabetes as a comorbid condition. This might be attributed to the higher age of the recruited patients since the majority were amongst the age group of 60 to 70 years, and along with age, the prevalence of such diseases also increases [28].

The quality of life in patients receiving cetuximab and cisplatin was high compared to the three-drug regimen. However, as per CSPOR HN02 trial, this three-drug regimen shows good overall survival (OS) and quality of life [29]. Similarly, in the TPEx trial, the three-drug combination which is comprised of docetaxel instead of paclitaxel has shown better outcome and quality of life [30]. These observations indicate that combination of taxanes along with cetuximab and platinum analogue has superior clinical outcomes and quality of life. However, its correlation with improvement in OS and progression-free survival must be established.

Both these regimens have shown similar safety profile; however, slight superiority was observed in cohort 2 who had received three-drug regimen. Additionally, these regimens demonstrated superior safety when compared to conventional treatment methods that used 5-FU [4]. None of the patient in this study experienced cardiac toxicity. The similar incidence was noted in case of febrile neutropenia in patients treated with cetuximab, cisplatin, and paclitaxel and patients treated with cetuximab, cisplatin, and 5-FU. In addition, none of the patient in our study developed grade 4 toxicities, whereas 31% of patients enrolled in EXTREME trial have reported grade 4 toxicities [4].

A few clinical trial had discovered that cetuximab might be combined with paclitaxel with possible compatibility and favorable efficacy following platinum loss, and that this combination could be more successful than the EXTREME regimen for the selected individuals [31]. The three-drug regimen utilized in this trial has shown superior quality of life compared to the combination of cetuximab, cisplatin, and 5-FU [23].

The results clearly show that the QALYs were significantly improved in cohort 2 compared to cohort 1 after completion of therapy. In the beginning, the steep decline in QALYs was observed which might be attributed to the first-time exposure of anticancer agents and their toxic effects. However, the QALYs were significantly improved after administration of the 3rd chemotherapy cycle. The three-drug regimen has shown significant improvement in QALYs at the end of therapy compared to two-drug regimens as well as EXTREME regimen.

However, the cetuximab-based three-drug regimen is category 2B evidence for the management of R/M HNSCC, and the preferred primary systemic therapy either alone or concurrent with radiation therapy is high-dose cisplatin alone or carboplatin and 5-FU infusion as per NCCN guidelines version 2.0 for head and neck cancer [32]. This is the major limitation of this article; however, in our study, the physician has prescribed the cetuximab-based three-drug regimen, and it has shown the significant improvement in patient’s health-related quality of life compared to two-drug regimen. Moreover, large cohort of R/M HNSCC, multiple treatment arms as per latest NCCN guideline, and the consideration of clinical outcomes and its correlation with survival need to be assessed for better clinical management.

### Recommendations for further research

Future work needs to explore the impact of different chemotherapeutic regimens for the management of R/M HNSCC on clinical and humanistic outcomes. Since the patients suffering from advance stage cancer have poor clinical prognosis, the quality of life remains an important treatment outcome. Therefore, the impact of chemotherapeutic regimen on humanistic outcome is important to analyze.

## Conclusion

The three-drug therapy consisting of cetuximab, cisplatin, and paclitaxel has shown significant improvement in patients’ QALYs compared to two-drug regimens of cetuximab and cisplatin. In terms of safety, we found that this three-drug regimen is superior than conventional three-drug regimen consisted of cetuximab, cisplatin, and 5-FU and quite similar to the two-drug regimen. This study is helpful for clinicians and patients to determine which regimen should be utilized based on its humanistic outcome in R/M HNSCC. Thus, if the therapy consisted of three-drug regimen is used instead of two-drug regimen, it will have a positive impact on humanistic outcome in recurrent or metastatic HNC patients.

## Supplementary Information

**Additional file 1.**

## Data Availability

The datasets generated during and/or analyzed during the current study are available from the corresponding author on reasonable request.

## Abbreviations

R/M HNSCC: Recurrent or metastatic head and neck squamous cell carcinoma
HNC: Head and neck cancer
QALYs: Quality-adjusted life-years
EGFR: Epidermal growth factor receptor
OS: Overall survival
ECOG: Eastern Cooperative Oncology Group

## References

[1] Khadela A, Vyas B. Assessment of the utilization pattern of chemotherapy agents in patients with head and neck cancer in an oncology hospital. Drugs Ther Perspect. 2020;36(7):303–9.

[2] Prabhash K, Babu G, Chaturvedi P, Kuriakose M, Birur P, Anand AK, et al. Indian clinical practice consensus guidelines for the management of squamous cell carcinoma of head and neck. Indian J Cancer. 2020;57(5):1. 32167063 10.4103/0019-509X.278971

[3] Price KA, Cohen EE. Current treatment options for metastatic head and neck cancer. Curr Treat Options Oncol. 2012;13(1):35–46. 22252884 10.1007/s11864-011-0176-y

[4] Vermorken JB, Mesia R, Rivera F, Remenar E, Kawecki A, Rottey S, et al. Platinum-based chemotherapy plus cetuximab in head and neck cancer. N Engl J Med. 2008;359(11):1116–27. 18784101 10.1056/NEJMoa0802656

[5] Baselga J, Trigo JM, Bourhis J, Tortochaux J, Cortés-Funes H, Hitt R, et al. Phase II multicenter study of the antiepidermal growth factor receptor monoclonal antibody cetuximab in combination with platinum-based chemotherapy in patients with platinum-refractory metastatic and/or recurrent squamous cell carcinoma of the head and neck. J Clin Oncol. 2005;23(24):5568–77. 16009950 10.1200/JCO.2005.07.119

[6] Argiris A, Karamouzis MV, Raben D, Ferris RL. Head and neck cancer. Lancet. 2008;371(9625):1695–709. 18486742 10.1016/S0140-6736(08)60728-X PMC7720415

[7] Cho BC, Keum KC, Shin SJ, Choi HJ, Lee YJ, Kim SH, et al. Weekly docetaxel in patients with platinum-refractory metastatic or recurrent squamous cell carcinoma of the head and neck. Cancer Chemother Pharmacol. 2009;65(1):27–32. 19381630 10.1007/s00280-009-0999-4

[8] Colevas AD. Systemic therapy for metastatic or recurrent squamous cell carcinoma of the head and neck. J Natl Compr Canc Netw. 2015;13(5):e37–48. 26158134 10.6004/jnccn.2015.0080

[9] Burtness B, Goldwasser MA, Flood W, Mattar B, Forastiere AA. Phase III randomized trial of cisplatin plus placebo compared with cisplatin plus cetuximab in metastatic/recurrent head and neck cancer: an Eastern Cooperative Oncology Group study. J Clin Oncol. 2005;23(34):8646–54. 16314626 10.1200/JCO.2005.02.4646

[10] Saif MW, Shah MM, Shah AR. Fluoropyrimidine-associated cardiotoxicity: revisited. Expert Opin Drug Saf. 2009;8(2):191–202. 19309247 10.1517/14740330902733961

[11] Bernad IP, Trufero JM, Urquizu LC, Pazo Cid R, de Miguel AC, Agustin M, et al. Activity of weekly paclitaxel− cetuximab chemotherapy in unselected patients with recurrent/metastatic head and neck squamous cell carcinoma: prognostic factors. Clin Transl Oncol. 2017;19(6):769–76. 28120324 10.1007/s12094-016-1604-z

[12] Posch D, Fuchs H, Kornek G, Grah A, Pammer J, Aretin M-B, et al. Docetaxel plus cetuximab biweekly is an active regimen for the first-line treatment of patients with recurrent/metastatic head and neck cancer. Sci Rep. 2016;6(1):1–7. 27597175 10.1038/srep32946 PMC5011715

[13] Guigay J, Even C, Mayache-Badis L, Debbah M, Saada-Bouzid E, Tao Y, et al. Long-term response in patient with recurrent oropharyngeal carcinoma treated with cetuximab, docetaxel and cisplatin (TPEx) as first-line treatment followed by cetuximab maintenance. Oral Oncol. 2017;68:114–8. 28347701 10.1016/j.oraloncology.2017.03.009

[14] Baselga J. The EGFR as a target for anticancer therapy—focus on cetuximab. Eur J Cancer. 2001;37:16–22. 10.1016/s0959-8049(01)00233-7 11597400

[15] Von Mässenhausen A, Braegelmann J, Billig H, Thewes B, Queisser A, Vogel W, et al. Implication of the receptor tyrosine kinase AXL in head and neck cancer progression. Int J Mol Sci. 2016;18(1):7. 28025482 10.3390/ijms18010007 PMC5297642

[16] Devlin NJ, Krabbe PF. The development of new research methods for the valuation of EQ-5D-5L. Eur J Health Econ. 2013;14 Suppl 1(Suppl 1):S1–3. 10.1007/s10198-013-0502-3 PMC3728454 23900659

[17] Dilla T, Lizan L, Paz S, Garrido P, Avendaño C, Cruz-Hernández JJ, et al. Do new cancer drugs offer good value for money? The perspectives of oncologists, health care policy makers, patients, and the general population. Patient Prefer Adherence. 2016;10:1. 26719677 10.2147/PPA.S93760 PMC4690649

[18] Wang SJ, Fuller CD, Choi M, Thomas CR Jr. A cost-effectiveness analysis of adjuvant chemoradiotherapy for resected gastric cancer. Gastrointest Cancer Res. 2008;2(2):57. 19259297 PMC2630819

[19] Sommers BD, Beard CJ, D'Amico AV, Kaplan I, Richie JP, Zeckhauser RJ. Predictors of patient preferences and treatment choices for localized prostate cancer. Cancer. 2008;113(8):2058–67. 18704993 10.1002/cncr.23807

[20] Kozminski MA, Neumann PJ, Nadler ES, Jankovic A, Ubel PA. How long and how well: oncologists’ attitudes toward the relative value of life-prolonging v. quality of life-enhancing treatments. Med Decis Making. 2011;31(3):380–5. 21088130 10.1177/0272989X10385847

[21] Khadela A, Vyas B, Bhikadiya V, Naik P. Impact of clinical pharmacist services on quality adjusted life years in head and neck cancer patients. Int J Clin Pharm. 2021;43(5):1208–17. 33528804 10.1007/s11096-021-01235-0

[22] Velentgas P, Dreyer NA, Nourjah P, Smith SR, Torchia MM, editors. Developing a Protocol for Observational Comparative Effectiveness Research: A User's Guide. Rockville: Agency for Healthcare Research and Quality (US); 2013. 23469377

[23] Zheng Y, Dou H, Li Q, Sun Y, Wang Y, Zhang W. Efficacy and safety of cetuximab plus cisplatin alone or in combination with paclitaxel in patients with head and neck squamous cell carcinoma: a randomized trial. Cancer Control. 2021;28:1073274821997444. 34029149 10.1177/1073274821997444 PMC8204453

[24] Grénman R, Chevalier D, Gregoire V, Myers E, Rogers S. Treatment of head and neck cancer in the elderly. Eur Arch Otorhinolaryngol. 2010;267(10):1619–21. 20454970 10.1007/s00405-010-1263-6

[25] Zhang Y, Wang R, Miao L, Zhu L, Jiang H, Yuan H. Different levels in alcohol and tobacco consumption in head and neck cancer patients from 1957 to 2013. PLoS One. 2015;10(4):e0124045. 25875934 10.1371/journal.pone.0124045 PMC4395416

[26] Negri E, Boffetta P, Berthiller J, Castellsague X, Curado MP, Maso LD, et al. Family history of cancer: pooled analysis in the International Head and Neck Cancer Epidemiology Consortium. Int J Cancer. 2009;124(2):394–401. 18814262 10.1002/ijc.23848 PMC3711193

[27] Chin D, Boyle GM, Porceddu S, Theile DR, Parsons PG, Coman WB. Head and neck cancer: past, present and future. Expert Rev Anticancer Ther. 2006;6(7):1111–8. 16831082 10.1586/14737140.6.7.1111

[28] Paleri V, Wight RG, Silver CE, Haigentz M Jr, Takes RP, Bradley PJ, et al. Comorbidity in head and neck cancer: a critical appraisal and recommendations for practice. Oral Oncol. 2010;46(10):712–9. 20850371 10.1016/j.oraloncology.2010.07.008

[29] Tahara M, Kiyota N, Yokota T, Hasegawa Y, Muro K, Takahashi S, et al. Phase II trial of combination treatment with paclitaxel, carboplatin and cetuximab (PCE) as first-line treatment in patients with recurrent and/or metastatic squamous cell carcinoma of the head and neck (CSPOR-HN02). Ann Oncol. 2018;29(4):1004–9. 29408977 10.1093/annonc/mdy040

[30] Guigay J, Fayette J, Dillies A, Sire C, Kerger J, Tennevet I, et al. Cetuximab, docetaxel, and cisplatin as first-line treatment in patients with recurrent or metastatic head and neck squamous cell carcinoma: a multicenter, phase II GORTEC study. Ann Oncol. 2015;26(9):1941–7. 26109631 10.1093/annonc/mdv268

[31] Peron J, Ceruse P, Lavergne E, Buiret G, Pham B-N, Chabaud S, et al. Paclitaxel and cetuximab combination efficiency after the failure of a platinum-based chemotherapy in recurrent/metastatic head and neck squamous cell carcinoma. Anticancer Drugs. 2012;23(9):996–1001. 22643048 10.1097/CAD.0b013e32835507e5

[32] NCCN Guidelines Head and Neck Cancers Version 2 [Available from: https://www.nccn.org/professionals/physician_gls/pdf/head-and-neck.pdf]. Accessed 7 Nov 2022.

