# Supplementary material for: Impact of cetuximab plus cisplatin alone and cetuximab plus cisplatin and paclitaxel regimen on humanistic outcome in head and neck cancer
Source: J Egypt Natl Canc Inst. 2023 Jan 19;35:1. doi: 10.1186/s43046-023-00160-9 (PMC13314003; doi:10.1186/s43046-023-00160-9)
Supplement: Supplementary file 1 — Additional file 1. [file 43046_2023_160_MOESM1_ESM.docx]

# Health Questionnaire

# English version for India

Under each heading, please tick the ONE box that best describes your health TODAY

**MOBILITY**

I have no problems in walking about ❑

I have slight problems in walking about ❑

I have moderate problems in walking about ❑

I have severe problems in walking about ❑

I am unable to walk about ❑

**SELF-CARE**

I have no problems in bathing or dressing myself ❑

I have slight problems in bathing or dressing myself ❑

I have moderate problems in bathing or dressing myself ❑

I have severe problems in bathing or dressing myself ❑

I am unable to bathe or dress myself ❑

**USUAL ACTIVITIES** *(e.g. work, study, housework,*

*family or leisure activities)*

I have no problems doing my usual activities ❑

I have slight problems doing my usual activities ❑

I have moderate problems doing my usual activities ❑

I have severe problems doing my usual activities ❑

I am unable to do my usual activities ❑

**PAIN / DISCOMFORT**

I have no pain or discomfort ❑

I have slight pain or discomfort ❑

I have moderate pain or discomfort ❑

I have severe pain or discomfort ❑

I have extreme pain or discomfort ❑

**ANXIETY / DEPRESSION**

I am not anxious or depressed ❑

I am slightly anxious or depressed ❑

I am moderately anxious or depressed ❑

I am severely anxious or depressed ❑

I am extremely anxious or depressed ❑

- We would like to know how good or bad your health is

10

0

20

30

40

50

60

80

70

90

100

5

15

25

35

45

55

75

65

85

95

The best health
 you can imagine

The worst health
 you can imagine

TODAY.

- This scale is numbered from 0 to 100.
- 100 means the best health you can imagine.
  0 means the worst health you can imagine.
- Mark an X on the scale to indicate how your health is TODAY.
- Now, please write the number you marked on the scale in the box below.

YOUR HEALTH TODAY =
